# Automated abdominal aortic calcification scoring via deep learning: a multi-center validation of LVLCRNet

**DOI:** 10.1186/s12880-025-02072-7

**Published:** 2025-12-01

**Authors:** Zhehao Zhang, Zhenhong Shao, Guotian Hu, Xiuchao He, Qingqing Lu, Yuning Pan

**Affiliations:** 1 Department of Radiology, The First Affiliated Hospital of Ningbo University, Ningbo, 315000 China; 2 Department of Radiology, Cixi City People’s Hospital, Ningbo, Zhejiang 315300 China

**Keywords:** Abdominal aortic calcification, Deep learning, Contrastive rank-aware

## Abstract

**Background:**

To develop and validate a deep learning model for automated quantification of abdominal aortic calcification scores (AACS) adhering to the Kauppila protocol, with multicenter clinical validation.

**Methods:**

This retrospective multicenter study analyzed 2,660 lateral lumbar/thoracoabdominal radiographs from four centers, partitioned into development (training: *n* = 1,478; validation: *n* = 423) and test cohorts (internal: *n* = 211; external: *n* = 157 from Center C and *n* = 391 from Center D). We proposed the Lumbar Vertebrae Localization-Contrastive Rank-Aware Network (LVLCRNet), incorporating automatic lumbar vertebrae localization, aortic region segmentation, and contrastive rank-aware network for ordinal classification. Comparative analyses against baseline network and Lumbar Vertebrae Localization Network were conducted using expert-annotated AACS as ground truth (GT), evaluated through Wilcoxon matched-paired signed-rank test, intraclass correlation coefficient (ICC), mean absolute error (MAE), coefficient of determination (R²), Bland-Altman analysis, and multiclass accuracy.

**Results:**

No significant difference was found between LVLCRNet and GT, whereas the baseline network showed significant deviations from GT across all cohorts (*p* < 0.017, Bonferroni-corrected). LVLCRNet achieved superior agreement with GT, demonstrating R² of 0.858 (internal) and 0.842/0.837 (external), ICC of 0.916 (internal) and 0.904/0.899 (external), and MAE of 1.547 (internal) and 1.189/1.972 (external). Bland-Altman analysis showed minimal systemic bias. Classification accuracy reached 82.94% (internal) and 79.62%/81.59% (external), outperforming comparators by 5.10–9.98%.

**Conclusion:**

LVLCRNet provides reliable automated AACS through integrated anatomic localization and contrastive rank-aware learning. Its strong generalizability and precision in severity grading support clinical utility for cardiovascular risk stratification.

**Supplementary Information:**

The online version contains supplementary material available at 10.1186/s12880-025-02072-7.

## Background

Abdominal Aortic Calcification (AAC) is one of the earliest vascular sites to develop calcification and serves as a marker of widespread atherosclerosis in other vascular beds. AAC exhibits a high prevalence, affecting approximately 33% of individuals aged 45–54 years and up to 90% of those over 75 years old [1, 2]. For older patients with type 2 diabetes mellitus or chronic kidney disease requiring dialysis, the prevalence ranges between 84% and 97% [3]. The severity of AAC has been established as an independent predictor of cardiovascular disease incidence and mortality. People with any or more advanced AAC had twice the relative risk and 9% to 17% absolute risk difference for cardiovascular events, fatal cardiovascular events, and all-cause mortality compared with those in the lowest reported AAC category [4–7]. Therefore, accurate and reproducible quantification of AAC severity is of utmost importance for patient management.

Endorsed by the KDIGO 2017 guidelines, lateral abdominal radiography serves as the clinical standard for AAC assessment, with the Kauppila scoring system being the predominant methodology due to its operational practicality and evidence-based validity [8–11]. The protocol systematically segments the aorta along lumbar vertebrae L1-L4 using intervertebral midpoints as anatomical landmarks. Calcification severity is graded 0–6 per vertebral segment, with summation yielding the AAC score (AACS) (range: 0–24). AACS has been shown to be associated with atherosclerosis in other vascular beds, as well as higher future risk of coronary, cerebrovascular and cardiovascular disease in older men and women [12, 13]. However, current AACS relies on manual visual assessment—a time-consuming approach prone to high interobserver variability and poor reproducibility due to inherent subjectivity [14]. Consequently, developing an automated and precise tool emerges as a critical clinical need to standardize assessments while reducing diagnostic variability and operator workload.

Deep learning-based convolutional neural networks (CNNs) have demonstrated promising performance in medical image analysis [15, 16]. In the context of automated AACS, some progress has been made in exploring these methods. Most of these methods treat the entire radiography as a single sample based on holistic regression calculations, while ignoring the relative rankings of the scoring system. This approach also disregards segment-specific scoring (L1–L4), leading to incomplete capture of calcification details in individual aortic segments. Additionally, deep learning models face persistent challenges from overfitting and dataset shift, necessitating external validation to ensure robust generalizability. Many deep learning algorithms exhibit marked performance degradation on external datasets [17]. Notably, most proposed automated AACS methods have been tested exclusively on internal datasets [18, 19], lacking guarantees of consistent, cross-institutional performance. Thus, there remains a critical need for methodologies that preserve the ordinal ranking and segment-specific characteristics of AACS while ensuring cross-institutional robustness.

This study introduces a novel lumbar vertebrae localization-based contrastive rank-aware network (LVLCRNet) for automated AACS prediction adhering to the Kauppila scoring protocol. The model’s technical contributions systematically align clinical scoring criteria with deep learning frameworks through three innovations: (1) Anatomical Grounding: A CNN-driven vertebral landmarks detection module precisely localizes L1-L4 segments, enabling region-specific calcification analysis anchored to standardized anatomical landmarks. (2) Ordinal Feature Regularization: The network ranks the samples in a batch according to their AACS and then contrasts them against each other based on their relative rankings. It mirrors Kauppila’s hierarchical scoring logic, preserving ordinal relationships between calcification severity grades. (3) Cross-Institutional Robustness: Validation across four independent datasets addresses apparatus variability and demographic heterogeneity, demonstrating generalizability beyond single-center training paradigms.

## Methods

### Data enrollment and allocation

This retrospective study was approved by institutional review boards at all participating centers (Approval No. 2025 No.040 A).

**Fig. 1:**
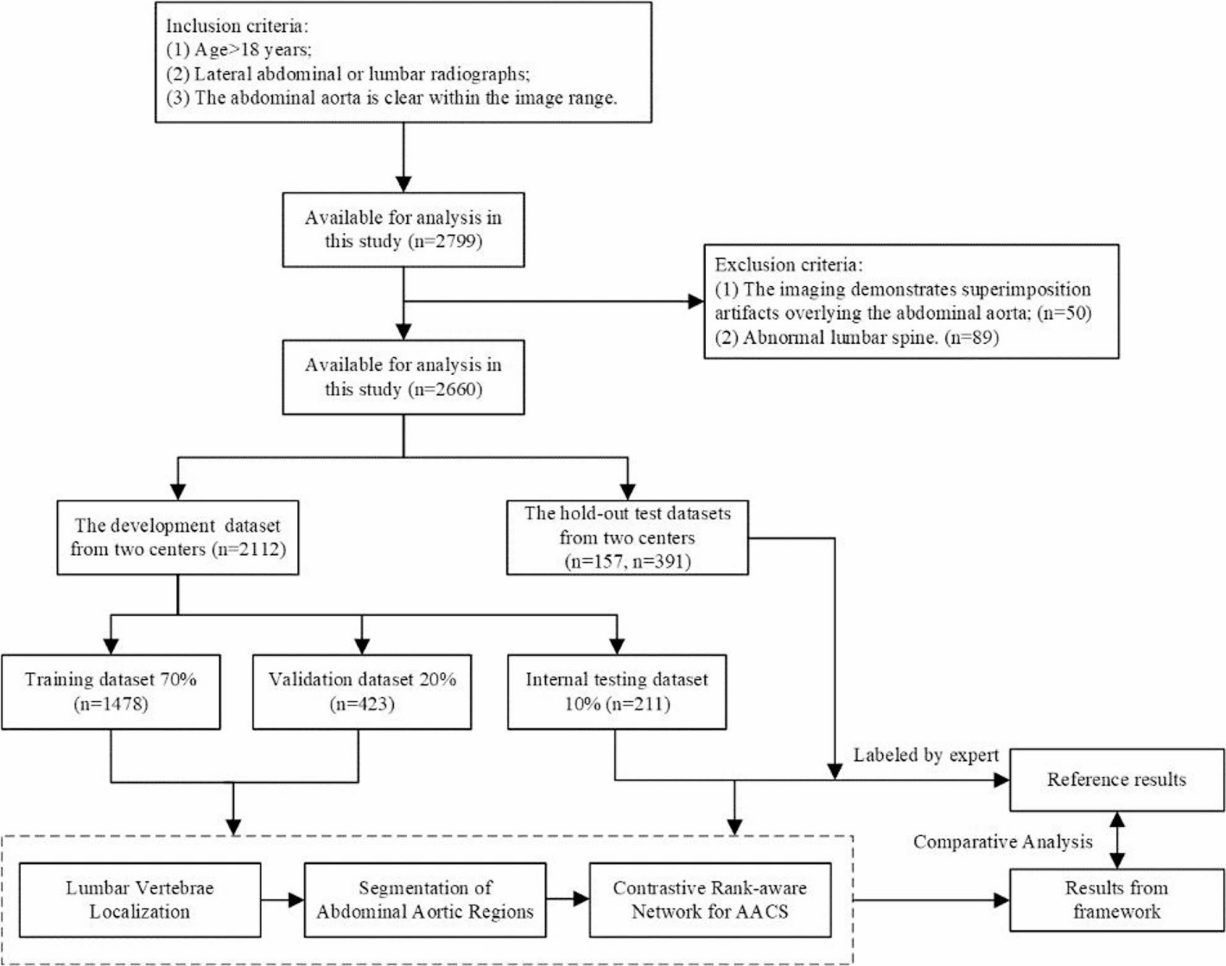
Flowchart of object inclusion and exclusion criteria for training, validation, Internal Test Set and External Test Sets

From August 2015 to October 2022, lateral lumbar/thoracoabdominal X-ray images were retrospectively collected from the Picture Archiving and Communication System of four hospitals. The inclusion criteria were: (a) patients aged ≥ 40 years; (b) radiographs demonstrating clear visualization of lumbar vertebrae (L1-L4). The exclusion criteria were: (a) overlap of the aortic region with radiopaque intestinal contents (due to medications or fecal stasis), (b) incomplete coverage of L1–L5 vertebrae or presence of vertebral deformities, (c) indistinguishable lumbar vertebral anatomy. Figure 1 shows the patient selection workflow for training, validation, internal test, and external test sets. Applying predefined criteria identified 2,799 eligible radiographs, with 139 excluded for protocol violations, resulting in a final analytic cohort of 2,660 radiographs. To ensure a rigorous evaluation and prevent data leakage, we note that each radiograph in our dataset originates from a distinct subject. A detailed summary of the imaging acquisition protocols and key technical parameters for each contributing center is provided in Supplementary Table 1S. Finally, 2,660 eligible radiographs were divided into two datasets based on the different center: the model development dataset (combined data from two high-volume centers, *n* = 2,112) and the external validation dataset (comprising radiographs from two independent institutions, *n* = 548). The development dataset was divided into training set, validation set, and internal test set in a ratio of 7:2:1.

### AACS manual evaluation

AACS was independently assessed by two radiologists with 6 and 8 years of experience, both blinded to patient clinical data. Discrepancies were resolved by a third physician with 15 years of cardiovascular imaging expertise. The scoring criteria for AACS encompassed vertebral levels L1–L4, with the anterior and posterior aortic walls evaluated respectively using each lumbar segment as a scoring unit. Score 0 indicates no calcification; score 1 indicates calcification affecting < 1/3 of segment length; score 2 indicates calcification involving 1/3 to 2/3 of segment length; score 3 indicates calcification covering >2/3 of segment length. Total AACS was calculated as the sum of all segment scores (range: 0–24). The reliability was quantified using the intraclass correlation coefficient (ICC) based on a two-way random-effects model for absolute agreement, single measures [ICC(2,1)]. The inter-reader reliability was strong across all sets: an ICC(2,1) of 0.913 (95% CI: 0.901 to 0.934) was achieved in the Center A and B, 0.919 (95% CI: 0.898 to 0.940) in Center C, and 0.922 (95% CI: 0.903 to 0.945) in Center D. Based on previously validated criteria, AAC was categorized into three severity groups: no/mild calcification (0 ≤ AACS ≤ 4), moderate calcification (4 < AACS ≤ 15), and severe calcification (15 < AACS ≤ 24) [20].

### Lumbar vertebrae annotation

Using LabelMe software, five lumbar vertebral bodies (L1–L5) underwent manual labeling with four corner landmarks per vertebra, specifically the upper-left, lower-left, upper-right, and lower-right corners. The annotation process was performed independently by two radiologists with 5 and 10 years of clinical experience, respectively. Final landmark coordinates were determined by averaging the measurements from both annotators to ensure consistency and reliability.

### Model architecture

**Fig. 2:**
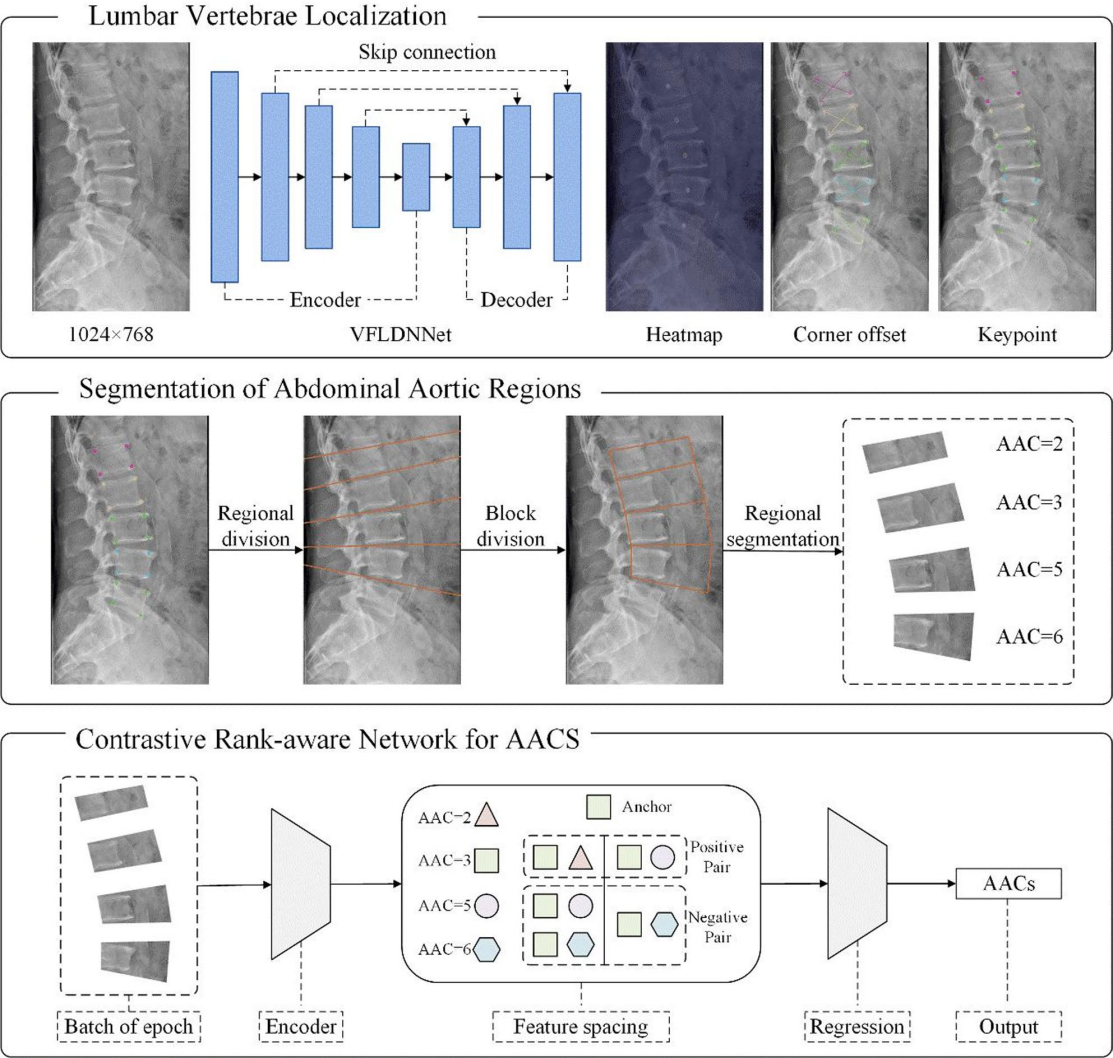
The overall architecture of LVLCRNet. In the Contrastive Rank-aware Network for AACS, when a sample with an AACS of 3 serves as the anchor, samples with a score of 2 (score distance = 1) form positive pairs, while samples with scores of 5 and 6 (score distance > 1) are selected as negative pairs. Conversely, when a sample with a score of 5 creates a positive pair with anchor, only samples with a score of 6 (score distance > 1) qualify as negative pairs. AACS = abdominal aortic calcification score

Our model consists of three steps: lumbar vertebrae localization, segmentation of the abdominal aorta region, and contrastive rank-aware network for AACS. The architecture proposed in this study is shown in Fig. 2.

#### Lumbar vertebrae localization

This process is rooted in deep learning-based landmark detection, leveraging Vertebra-Focused Landmark Detection Network (VFLDNet) specifically designed for scoliosis assessment [21]. The VFLDNet architecture consists of an encoder, decoder, and multi-output branches. Lateral radiographs are processed through a stacked CNN-based encoder-decoder framework, where the network generates center heatmap, center offset and corner offset. After decoding these outputs (detailed process see [21]), we extracted 20 precise anatomical landmarks corresponding to the lumbar vertebrae.

#### Segmentation of abdominal aortic regions

To simulate the Kauppila scoring, we performed vertebral level-based segmentation of the abdominal aorta using anatomical landmarks, as illustrated in Fig. 2. First, the midpoints between adjacent vertebrae were calculated and connected to define aortic segment boundaries. To address incomplete visualization of the T12 vertebra in some radiographs, we approximated the aortic boundary at the L1 level by connecting the upper-left and upper-right landmarks of L1. After determining five primary boundaries, each was empirically extended abdominally by a distance matching its original length. Finally, adjacent extended lines were connected at their start and end points to delineate four distinct abdominal aortic segments (AAS).

#### Contrastive rank-aware network for AACS

Based on ResNet101 [22], the input consists of AAS and the output is the AACS for each segment. To capture the ordinality of AAS, we introduced a contrastive rank-aware loss in addition to the regression loss [Mean Absolute Error (MAE)]. This strategy orders the distances in the embedding space based on the AACS, and then leverages it to predict the AACS. Specifically, for AAS $\:{v}_{i}$, $\:{v}_{j}$, $\:{v}_{k}$, if $\:{S}_{i,j}=\left\{{F}_{k}\right|k\ne\:i,d\left({v}_{i},{v}_{k}\right)>d({v}_{i},{v}_{j})\}$, the model enforces $\:d{\prime\:}({F}_{i},{F}_{k})>d{\prime\:}({F}_{i},{F}_{j})$, where $\:{v}_{i}$ serves as the anchor, $\:({v}_{i},{v}_{j})$ as the positive pair, and $\:({v}_{i},{v}_{k})$ as the negative pair. Here, $\:d(\cdot,\cdot)$ denotes the L1-distance between AACS of two AAS, $\:d{\prime\:}(\cdot,\cdot)$ denotes the L2-distance between feature vectors of two AAS and *F* represents the feature vector of a sample extracted by the ResNet101 backbone. The optimization objective is defined as follows:

$$\begin{aligned}\:{L}_{CR}&=\frac{1}{N}\sum\limits_{i=1}^{N}\frac{1}{N-1}\sum\limits_{j=1,j\ne\:i}^{N}\cr&\quad-log\frac{\text{e}\text{x}\text{p}\left(sim\left({F}_{i},{F}_{j}\right)/\tau\:\right)}{\sum\nolimits_{{F}_{k}}\text{e}\text{x}\text{p}\left(sim\left({F}_{i},{F}_{k}\right)/\tau\:\right)}\end{aligned}$$

Where *N* is batch size. $\:sim\left(a,b\right)$ is a similarity measurement function, which adopts the cosine similarity. *τ* is a scaling hyper-parameter. For anchor sample $\:{v}_{i}$, compare any other sample $\:{v}_{j}$ in the batch with it, forcing the feature similarity between $\:{v}_{i}$ and $\:{v}_{j}$ to be greater than the feature similarity between $\:{v}_{i}$ and any other AAS $\:{v}_{k}$ in the batch. Finally, after obtaining the four AAS level scores for each radiograph, we add them up to obtain the sample level AACS.

### Data preprocessing and training protocol

During the lumbar vertebrae landmark detection phase, radiographs were uniformly resized to 1024 × 786 pixels using isotropic scaling combined with zero-padding. For the AACS prediction phase, cropped X-ray images were standardized to 256 × 786 pixels to focus on the segmented aortic regions. All the methods are implemented in PyTorch and trained with 200 epochs on an NVIDIA RTX 4090 GPU. During the training process, the initial learning rate is set to 0.001, and the learning rate decreases by 10 times every 50 epochs. The weight initialization parameters are set using the pretraining parameters provided with PyTorch. And we use image augmentation methods such as image flipping, brightness, and contrast adjustment.

### Comparison with other methods

To elucidate the superiority of LVLCRNet, a comparative analysis is conducted with other methods. The methods under comparison are simply delineated below: (1) holistic regression-based network (HRNet): This approach did not involve lumbar vertebrae localization or segmentation of abdominal aortic regions. ResNet101 was used directly to predict the abdominal aortic calcification score (AACS) from the radiographs. (2) LVLCRNet w/o rank (LVLNet): This model shared the same lumbar vertebrae localization and abdominal aortic region segmentation processes as the full LVLCRNet, but did not incorporate the vertebral ranking strategy.

### Statistical analysis

Statistical analyses were conducted using SPSS 27.0, with continuous variables expressed as mean ± standard deviation for normally distributed data or median and interquartile range (IQR) for non-normally distributed data, and categorical variables presented as frequencies and percentages. For lumbar vertebrae landmark detection, we computed the MAE for each keypoint between the predictions of *LVLNet/*LVLCRNet and the manual labels provided by the two radiologists. In AAC prediction analysis, the final AACS of each sample are obtained by adding the predicted AACS of AAS. AACS comparisons among methods were assessed via paired t-test or Wilcoxon matched-paired signed-rank test (Bonferroni-corrected) *and Two-One-Sided Tests (Δ = ±1 and ± 0.5 point)*, while agreement between automated predictions and manual scores was quantified using MAE, Bland-Altman analysis, and ICC with absolute agreement two-way random model (single measure). The Spearman rank correlation measured associations between predicted and ground truth (GT) AACS, R² evaluated regression model fit. To further address the comparative performance of the methods, we conducted a detailed paired difference analysis (model- GT). The severity of AAC was evaluated in three categories as previously mentioned. Boundary distance error (BDE) evaluates the distance to the correct category boundary when a sample is misclassified. Classification performance was evaluated using accuracy (ACC), sensitivity (SN), specificity (SP), positive predictive value (PPV), and negative predictive value (NPV). Significance thresholds were *P* < 0.017 for multiple comparisons (Bonferroni-corrected) and *P* < 0.05 for others.

### Implementation details

All models are implemented in PyTorch and trained for 200 epochs using an NVIDIA RTX 4090 GPU. The training process employs the Adam optimizer with an initial learning rate of 0.001, which is reduced by a factor of 0.5 every 50 epochs. To enhance the model’s generalization ability, data augmentation techniques are applied, including random horizontal flipping, brightness-contrast adjustment, and photometric augmentation. The full radiographs are resized to 1024 × 512 pixels using bilinear interpolation and input with a batch size of 8. The AACS images are resized to 384 × 512 pixels using the same interpolation method and trained with a batch size of 24.

## Results

### Data set characteristics

A total of 2660 subjects (median age, 67 years [IQR 60–75]; 1335 [50.2%] female; median AACS, 8 points [IQR 4–12]) were allocated to the development (training: *n* = 1,478; validation: *n* = 423) and Internal Test (*n* = 211) sets. External test included two cohorts: External Test Set 1 (*n* = 157 from Center C; 65 years [IQR 59–74], 46 [29.3%] female), and External Test Set 2 (*n* = 391 from Center D; 62 years [IQR 54–70], 181 [46.3%] female). No significant differences were observed in clinically relevant parameters across cohorts, except for sex distribution in Center C (*p* < 0.001). Detailed demographic characteristics are provided in Table 1.

**Table 1:** Subject demographic characteristics in the data sets

Characteristic	Center A and B			Center C	Center D	Total (*n* = 2660)	*P* Value
	Training (*n* = 1478)	Validation (*n* = 423)	Internal test (*n* = 211)	External test set 1 (*n* = 157)	External test set 2 (*n* = 391)		
Sex^a^							< 0.001
Female	777 (52.57%)	226 (53.43%)	105 (49.76%)	46 (29.30%)^*^	181 (46.29%)	1335 (50.19%)	
Male	701 (47.43%)	197 (46.57%)	106 (50.23%)	111 (70.70%)^*^	210 (53.71%)	1325 (49.81%)	
Age^b^	70 (63,77)	71 (64,77)	69 (63,76)	65 (59,74)	62 (54,70)	67 (60,75)	0.149
AACS^b^							
Overall	8 (5,12)	9 (5,13)	7 (4,11)	6 (3,10)	10 (5,14)	8 (4,12)	0.649
L1 segment	1 (0,2)	1 (0,2)	1 (0,2)	0 (0,2)	1 (0,3)	1 (0,2)	0.345
L2 segment	1 (0,2)	2 (1,3)	1 (0,2)	1 (0,2)	2 (1,3)	1 (0,3)	0.604
L3 segment	2 (1,4)	3 (2,4)	2 (1,4)	2 (1,4)	3 (2,4)	2 (1,4)	0.815
L4 segment	3 (2,4)	3 (2,4)	3 (1,5)	2 (1,4)	3 (2,5)	3 (2,4)	0.851
Severity^a^							0.147
No/Low	345 (23.34%)	77 (18.20%)	54 (25.59%)	53 (33.76%)	87 (22.25%)	616 (23.16%)	
Moderate	970 (65.63%)	290 (68.56%)	134 (63.51%)	85 (54.14%)	228 (58.31%)	1707 (64.17%)	
High	163 (11.03%)	56 (13.24%)	23 (10.90%)	19 (12.10%)	76 (19.43%)	337 (12.67%)	

^a^Number of patients (%), ^b^Median (interquartile range), ^*^Statistically significant

AACS = abdominal aortic calcification score

### Performance of lumbar vertebrae localization

The MAE of lumbar vertebral landmark localization was 18.47 pixels (2.48 mm) for the Internal Test Set and 16.58 pixels (2.23 mm) and 19.36 pixels (2.60 mm) for the two External Test Sets, respectively. Relative to the average dimensions of the vertebral bodies in the images (length: 279.58 pixels (38.58 mm); width: 198.32 pixels (27.37 mm), all errors remained below 7% of length (19.36/279.58 ≈ 6.9%) and 10% of width (19.36/198.32 ≈ 9.8%), demonstrating accuracy in clinical practice.

### Comparison of three methods

**Fig. 3:**
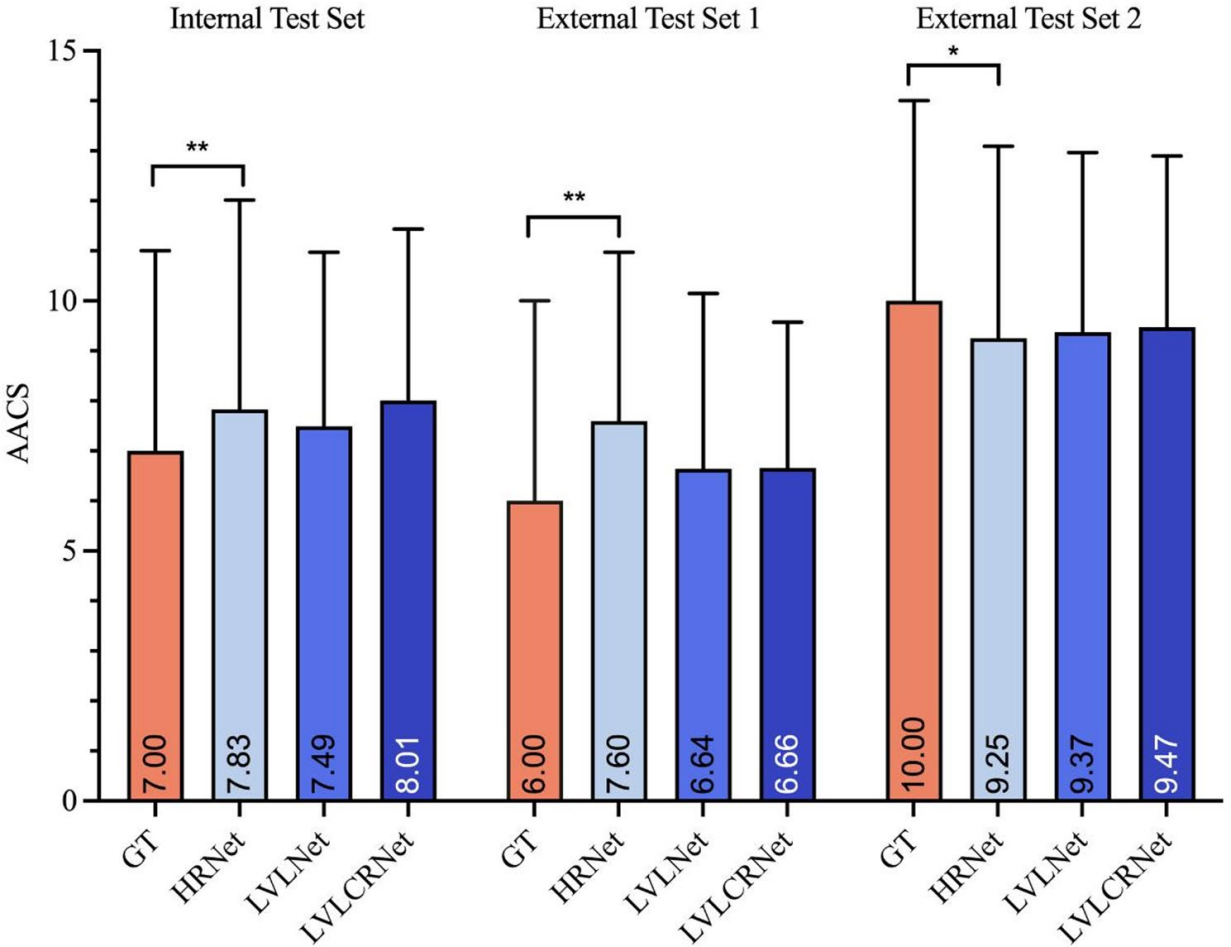
Comparison of AACS between three methods and GT. HRNet showed significant discrepancies from GT (*P* ≤ 0.005), while both LVLNet and LVLCRNet demonstrated no statistically significant differences compared to GT after Bonferroni correction (*P* > 0.017). AACS are presented as median values with interquartile ranges. Symbols: ** denotes *P* < 0.001; * denotes *P* = 0.005. AACS = abdominal aortic calcification score, GT = ground truth

The HRNet exhibited significant deviations from GT across all cohorts (*p* < 0.017, Bonferroni-corrected), while other methods demonstrated statistical equivalence (*p* ≥ 0.017) as shown in Fig. 3. Specifically, as detailed in Supplementary Table 2S, which showed the mean (0.32–0.62 point) and median (0.58–1.20 point) differences between HRNet and GT, this regression model exhibited a consistent overestimation bias across all three datasets. *The results of Two-One-Sided Tests demonstrated clinical equivalence (difference ≤ 1 AACS point) for all three models in most comparisons*,* except for HRNet in External Test 1. At the stricter ± 0.5 point margin*,* only LVLCRNet maintained statistical equivalence across all cohorts*,* demonstrating superior precision over both LVLNet and HRNet (see Supplementary* Table 3S *and* Fig. 1S*).*

**Fig. 4:**
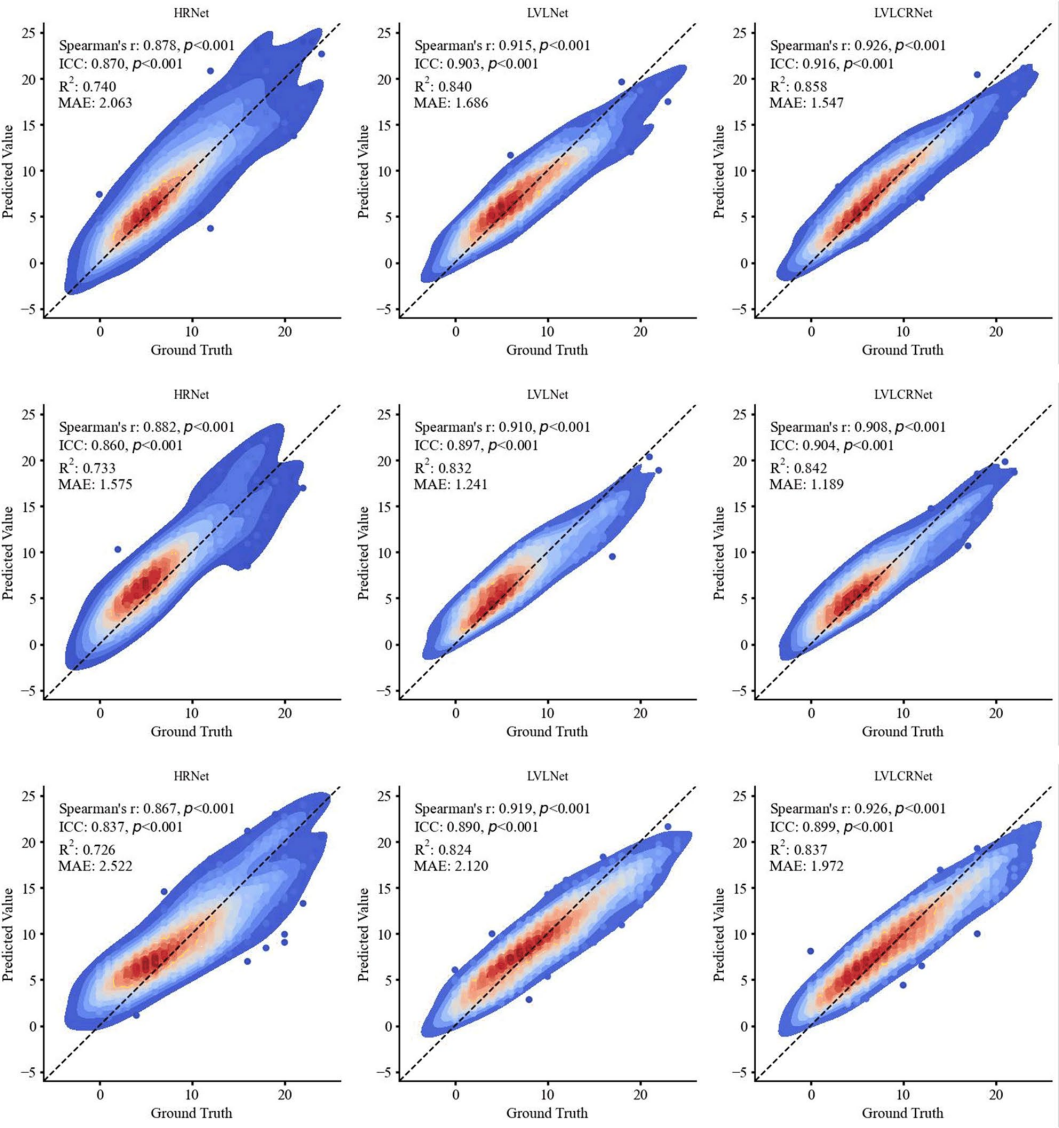
Scatter density plots comparing ground truth versus predicted AACS across three methods. Rows correspond to the Internal Test Set (top), External Test Set 1 (middle), and External Test Set 2 (bottom). Point density is visualized using a color gradient, with red indicating high density and blue indicating low density, reflecting the agreement distribution between manual and automated scores. AACS = abdominal aortic calcification score, ICC = intraclass correlation coefficient, MAE = mean absolute error

As summarized in Supplementary Table 2S, the mean differences reveal that LVLCRNet achieved the smallest deviation from the GT across all test sets, including the internal set and two external validation sets, with LVLNet consistently ranking second. Analysis of the median differences yielded a consistent finding: both LVLNet and LVLCRNet demonstrated superior agreement with the GT (i.e., smaller median differences) compared to HRNet on the Internal Test Set and External Test Set 1. Despite demonstrating robust correlations with GT scores, the integration of vertebral localization significantly enhanced model performance across all test sets, as evidenced by improvements in R² values (ΔR² = +0.098–0.100) and ICC (ΔICC = + 0.033–0.053). Notably, our proposed rank-aware method achieved the best R² values (0.858 [Internal Test Set], 0.842 [External Test Set 1], 0.837 [External Test Set 2]), ICCs (0.916 [Internal Test Set], 0.904 [External Test Set 1], 0.899 [External Test Set 2]) and MAE (1.547 [Internal Test Set], 1.189 [External Test Set 1], 1.972 [External Test Set 2]) demonstrating superior alignment with manual scoring outcomes (Table 2; Fig. 4).

**Table 2:** Detailed performance metrics comparison of all models on internal and external test sets

Test set	Model	Spearman’s *r* (95% CI)	ICC (95% CI)	*R*^2^ (95% CI)	MAE (95% CI)
Internal Test Set	HRNet	0.878 (0.832, 0.912)	0.870 (0.834, 0.899)	0.740 (0.660, 0.802)	2.063 (1.831, 2.307)
	LVLNet	0.915 (0.884, 0.935)	0.903 (0.877, 0.923)	0.840 (0.799, 0.871)	1.686 (1.520, 1.872)
	LVLCRNet	**0.926 (0.898**,** 0.944)**	**0.916 (0.893**,** 0.933)**	**0.858 (0.822**,** 0.884)**	**1.547 (1.387**,** 1.726)**
External Test Set 1	HRNet	0.882 (0.832, 0.917)	0.860 (0.820, 0.892)	0.733 (0.648, 0.792)	1.575 (1.411, 1.803)
	LVLNet	**0.910 (0.872**,** 0.932)**	0.897 (0.865, 0.920)	0.832 (0.785, 0.867)	1.241 (1.156, 1.407)
	LVLCRNet	0.908 (0.867, 0.934)	**0.904 (0.874, 0.926)**	**0.842 (0.794**,** 0.877)**	**1.189 (1.092**,** 1.360)**
External Test Set 2	HRNet	0.867 (0.838, 0.890)	0.837 (0.806, 0.863)	0.726 (0.681, 0.764)	2.522 (2.317, 2.682)
	LVLNet	0.919 (0.900, 0.933)	**0.890 (0.872, 0.905)**	0.824 (0.797, 0.847)	2.120 (1.900, 2.178)
	LVLCRNet	**0.926 (0.909**,** 0.938)**	**0.899 (0.882, 0.914)**	**0.837 (0.812**,** 0.858)**	**1.972 (1.785**,** 2.064)**

Notes: The optimal values are highlighted in bold

ICC = intraclass correlation coefficient, MAE = mean absolute error, CI = confidence interval

**Fig. 5:**
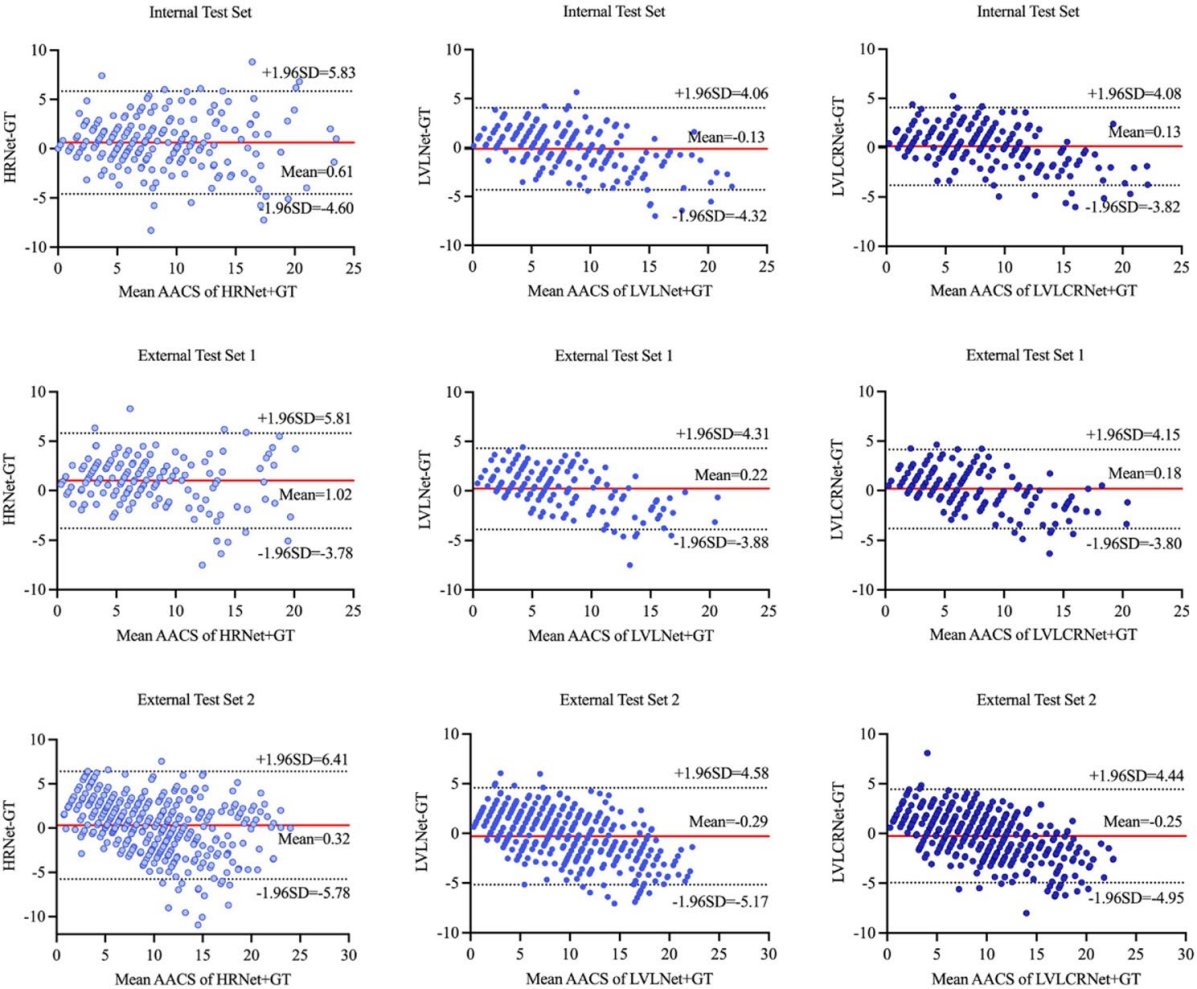
Bland-Altman plots assessing agreement in AACS between GT and three methods: HRNet, LVLNet, and LVLCRNet. Top row: Internal Test Set (*n* = 211); Middle row: External test set 1 (*n* = 157); Bottom row: External Test Set 2 (*n* = 391). Solid horizontal lines indicate mean differences (model - GT), with dotted lines marking 95% Limits of Agreement (± 1.96 SD). AACS = abdominal aortic calcification score, GT = ground truth, SD = Standard deviation

Furthermore, LVLCRNet consistently demonstrated smaller mean (SD) differences and narrower limits of agreement across all test sets, particularly in External Test Set 1 [LVLCRNet: 0.180 (2.028) point, ± 1.96SD (-3.795, 4.154); LVLNet: 0.219 (2.089) point, ± 1.96SD (-3.877, 4.314); HRNet: 1.017 (2.448) point, ± 1.96SD (-3.780, 5.814)] as shown in Fig. 5.

**Fig. 6:**
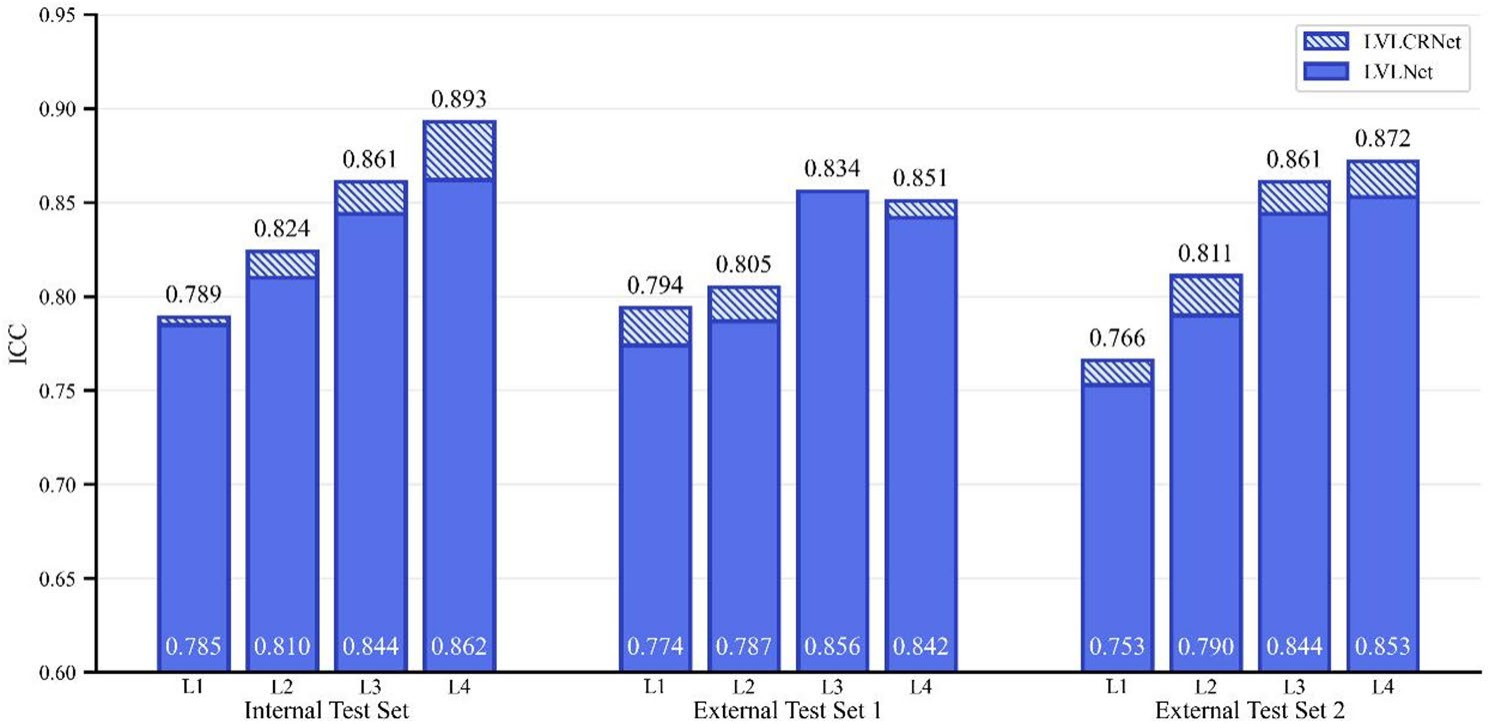
Comparison of ICCs between LVLCRNet (upper portion of bars) and LVLNet (lower portion of bars) across AAS L1-L4. From left to right: Internal Test Set, External Test Set 1, and External Test Set 2. Stacked bars represent segment-specific agreement with ground truth AACS. ICC = intraclass correlation coefficient, AAS = abdominal aortic segments, AACS = abdominal aortic calcification score

Following segment-specific ICC analysis, the integration of the rank-aware method improved ICC across all segments, except for the L3 segment in External Test Set 1 (Fig. 6). Furthermore, ICC exhibited a distal-to-proximal gradient (L4 > L3 > L2 > L1), with higher reliability observed in lower abdominal aortic regions (L3-L4) compared to upper segments (L1-L2).

### Performance comparison in severity classification

**Fig. 7:**
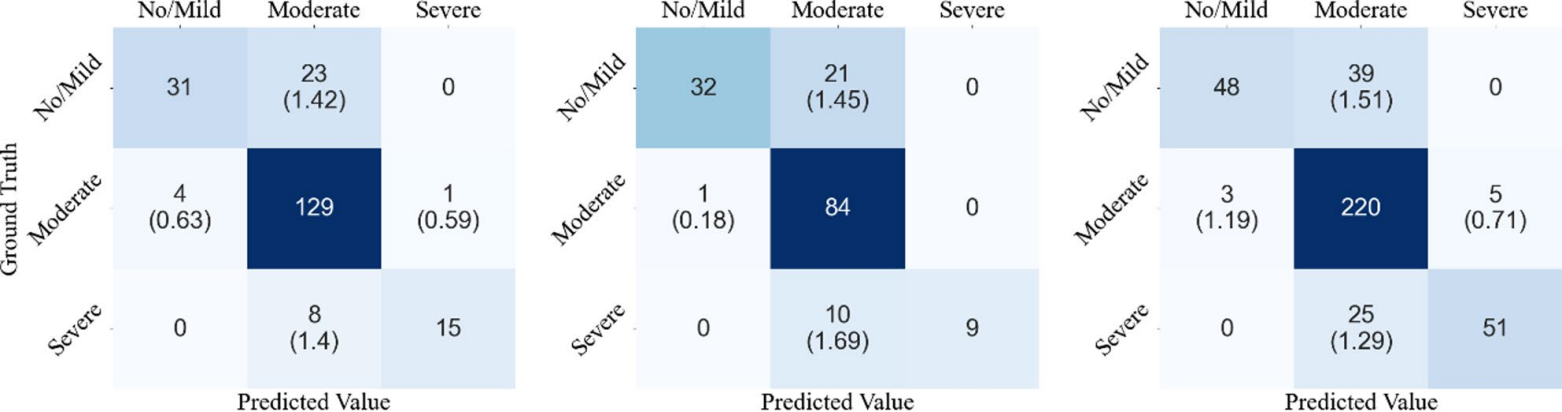
Confusion matrices of LVLCRNet across different test sets: Internal Test Set (left), External Test Set 1 (middle), and External Test Set 2 (right). For misclassified samples, the BDE is reported in parentheses, which quantifies the positional deviation between the predicted AACS and ground truth calcification boundaries. BDE = boundary distance error, AACS = abdominal aortic calcification score

As shown in Table 3, LVLCRNet demonstrated consistent superiority across all test cohorts, achieving the highest accuracy (ACC: 82.94% [Internal Test Set], 79.62% [External Test Set 1], 81.59 % [External Test Set 2]), specificity (SP: 85.33–86.05%), and predictive values (PPV/NPV: 87.55–90.00%/91.01–91.31%) compared to baseline models (HRNet/LVLNet). The rank-aware module improved sensitivity versus LVLNet (ΔSN = + 2.11–6.03%) and accuracy (ΔACC = + 1.90–3.82%), while sensitivity gains over HRNet were restricted to External Test Set 2 (72.92% vs. 60.95%, Δ=+11.97%). Although LVLCRNet tends to overestimate the no/mild class and underestimate the severe class, the mean BDE is not significant (mean BDE < 2) as shown in Fig. 7.

**Table 3:** Comparison of classification performance in severity evaluation in different test sets

	Model	ACC	SN	SP	PPV	NPV
Internal Test Set	HRNet	77.73	72.17	84.30	73.40	85.33
	LVLNet	81.04	70.86	84.04	85.87	89.68
	LVLCRNet	**82.94**	**72.97**	**85.56**	**87.65**	**91.01**
External Test Set 1	HRNet	74.52	**69.51**	82.58	80.15	86.80
	LVLNet	75.80	62.83	82.55	88.66	89.90
	LVLCRNet	**79.62**	68.86	**85.33**	**90.00**	**91.31**
External Test Set 2	HRNet	71.61	60.95	79.37	78.02	84.01
	LVLNet	79.28	70.49	84.69	83.97	89.26
	LVLCRNet	**81.59**	**72.92**	**86.05**	**87.55**	**91.20**

Notes: Maximum values are highlighted in bold

ACC = accuracy, SN = Sensitivity, SP = Specificity, PPV = positive predictive value, NPV = negative predictive value

## Discussion

Our study presents LVLCRNet, a novel deep learning framework for automated prediction of the AACS that integrates lumbar vertebrae localization and rank-aware ordinal regression. By simulating the Kauppila scoring protocol, our model achieved robust performance across multi-center datasets, demonstrating significant clinical applicability.

KDIGO 2017 recommend lateral abdominal radiography as a primary modality for detecting AAC in clinical practice, particularly for cardiovascular risk stratification in high-risk populations like dialysis patients [9]. While prior studies have predominantly focused on dual-energy X-ray absorptiometry (DXA), demonstrating promising technical performance, their clinical generalizability remains constrained. For instance, Sethi et al. developed a deep learning regression model for AACS on DXA images, achieving MAE of 1.27 [14]. However, their dataset was skewed toward mild calcifications (mean AACS: 1.47, ours median: 8), with limited representation of severe cases, thereby restricting insights into model performance across the full spectrum of calcification severity. Similarly, Reid et al. reported a CNN-based model on DXA-derived vertebral fracture assessment scans with comparable accuracy to our internal results (R²=0.86 vs. 0.858) [19]. In contrast, CT-based approaches, exemplified by Graffy et al., leverage high-resolution imaging for precise Agatston scoring via instance segmentation networks [23]. While CT remains the gold standard for volumetric calcification analysis, its reliance on specialized equipment and higher radiation exposure limits routine clinical adoption, particularly in resource-constrained settings.

Unlike prior studies that directly regressed global AACS from full images [19, 24–27], our method explicitly segments abdominal aortic regions based on lumbar vertebral landmarks, aligning with clinical workflows. In addition, segmented evaluation of AACS is more helpful in reducing the influence of background and capturing finer calcifications in smaller areas. Our experimental results indicate that significant improvements were achieved after using the segmentation method. Similarly, Wang et al. segmented Vertebrae T12-L5 and Aortic Walls on X-rays [18]. However, our framework uniquely leverages landmark detection based on deep learning, which is more labor-saving compared to previous annotation work, but still achieves higher R² (0.858 vs. 0.82) Notably, both studies observed improved performance in distal aortic segments (e.g., L4), likely due to reduced overlap with intestinal contents and clearer calcification visibility. Additionally, Saleem et al. introduced ordinal loss for AAC scoring on vertebral fracture assessment scans, our method optimizes this sequential relationship in AAS [24]. Our experiment shows that after introducing the -aware method, higher ICC and R^2^ were obtained. The rank-aware method enhances the consistency of feature expression for similar samples of AACS and the differences in feature expression for no similar samples by mining the ranking information of AACS and the continuity pattern of their feature expression.

Our analysis revealed distinct patterns in estimation bias across models and test sets (Supplementary Table 2S). HRNet consistently overestimated AACS values in all datasets, likely due to its whole-image architecture, which may misclassify dense structures such as bone as calcifications. In contrast, our LVLNet and LVLCRNet models exhibited bias patterns that correlated with test set characteristics. They showed minimal bias on the Internal Test Set, slight overestimation on External Test Set 1 (which has a lower median AACS of 6, indicating milder cases), and consistent underestimation on External Test Set 2 (with a higher median AACS of 10, indicating more severe cases). Given that the training set had a median AACS of 8, the models tend to overestimate in milder test sets and underestimate in more severe ones, reflecting a regression toward the training distribution.

Our model demonstrated robust overall performance in stratifying AAC severity but exhibited suboptimal SN, particularly in distinguishing severe calcification cases. This limitation aligns with findings from Sharif et al. who reported SN values of 60.4–74.2 across diverse datasets for AAC grading on DXA images [25]. Analysis of the confusion matrix revealed a systematic bias: our model tended to overestimate no/mild cases and underestimate severe cases, likely attributable to class imbalance, as the moderate calcification group dominated the training data (*n* = 970 vs. *n* = 345 [no/mild] and *n* = 163 [severe]). Such imbalance may skew feature learning toward the majority class, reducing sensitivity to underrepresented severity extremes—a common challenge in medical imbalanced datasets. Notably, our findings contradict those of Gilani et al. [28], who reported poorer classification performance for moderate AAC groups. This discrepancy may stem from divergent threshold definitions for severity stratification (no/mild: 0–1, moderate 2–5. severe ≥ 6 for them). Additionally, differences in imaging modalities (conventional X-ray vs. DXA) may further explain these variations.

Our study has several limitations that warrant consideration. First, the class imbalance in the three-category dataset (no/mild, moderate, and severe AAC) may constrain the generalizability of results, particularly for underrepresented severity groups (e.g., severe calcifications). Second, the abdominal aortic localization strategy, which relied on vertebral landmarks with empirical adjustments, lacks the precision of advanced segmentation techniques (e.g., pixel-wise annotation or deformable models), potentially introducing positional errors in segment-specific scoring. Thirdly, our framework only focuses on segmental AAC features while ignores the calcification pattern of the entire abdominal aorta, which may result in the loss of some meaningful information. Finally, the current work prioritizes automated AAC scoring via the Kauppila system but does not incorporate complementary clinical or biochemical data (e.g., renal function markers, lipid profiles), limiting its utility as a standalone cardiovascular risk predictor.

## Conclusions

The proposed LVLCRNet demonstrates robust performance in automated AAC assessment, achieving strong agreement with GT manual scores. By integrating anatomic localization and contrastive rank-aware regression, our framework addresses critical limitations of conventional regression-based methods, particularly in preserving the ordinal relationships inherent to calcification severity grades. The model’s generalizability across multi-center datasets and its superior performance in severity calcification underscore its potential as a clinically viable tool for cardiovascular risk stratification.

## Supplementary Information

Below is the link to the electronic supplementary material.

Supplementary Material 1

## Data Availability

Due to privacy restrictions, the datasets presented in this article are not publicly available. Requests to access the datasets should be directed to corresponding author.

## Abbreviations

AAC: Abdominal aortic calcification
AACS: Abdominal aortic calcification score
KDIGO: Kidney Disease: Improving Global Outcomes
CNN: Convolutional neural network
AAS: Abdominal aortic segments
LVLNet: Lumbar vertebrae localization-based network
LVLCRNet: Lumbar vertebrae localization-based contrastive rank-aware network
VFLDNet: Vertebra-Focused Landmark Detection Network
IQR: Interquartile range
MAE: Mean absolute error
ICC: Intraclass correlation coefficient
BDE: Boundary distance error
GT: Ground truth
ACC: Accuracy
SN: Sensitivity
SP: Specificity
PPV: Positive predictive value
NPV: Negative predictive value
DXA: Dual-energy X-ray absorptiometry

## References

[1] Wong ND, Lopez VA, Allison M, Detrano RC, Blumenthal RS, Folsom AR, et al. Abdominal aortic calcium and multi-site atherosclerosis: the multiethnic study of atherosclerosis. Atherosclerosis. 2011;214:436–41. 21035803 10.1016/j.atherosclerosis.2010.09.011 PMC3040451

[2] Ni G, Jia Q, Li Y, Cheang I, Zhu X, Zhang H, et al. Association of life’s essential 8 with abdominal aortic calcification and mortality among middle-aged and older individuals. Diabetes Obes Metabolism. 2024;26:5126–37. 10.1111/dom.15854 39165042

[3] Tatami Y, Yasuda Y, Suzuki S, Ishii H, Sawai A, Shibata Y, et al. Impact of abdominal aortic calcification on long-term cardiovascular outcomes in patients with chronic kidney disease. Atherosclerosis. 2015;243:349–55. 26519631 10.1016/j.atherosclerosis.2015.10.016

[4] Leow K, Szulc P, Schousboe JT, Kiel DP, Teixeira-Pinto A, Shaikh H, et al. Prognostic value of abdominal aortic calcification: A systematic review and Meta‐Analysis of observational studies. JAHA. 2021;10:e017205. 33439672 10.1161/JAHA.120.017205 PMC7955302

[5] Bastos Gonçalves F, Voûte MT, Hoeks SE, Chonchol MB, Boersma EE, Stolker RJ, et al. Calcification of the abdominal aorta as an independent predictor of cardiovascular events: a meta-analysis. Heart. 2012;98:988–94. 22668866 10.1136/heartjnl-2011-301464

[6] Wilson PWF, Kauppila LI, O’Donnell CJ, Kiel DP, Hannan M, Polak JM, et al. Abdominal aortic calcific deposits are an important predictor of vascular morbidity and mortality. Circulation. 2001;103:1529–34. 11257080 10.1161/01.cir.103.11.1529

[7] Orces CH. Abdominal aorta calcification identified on DXA scans and the risk of mortality in adults. J Bone Metab. 2024;31:236–45. 39307524 10.11005/jbm.2024.31.3.236 PMC11416878

[8] KDIGO 2017 clinical practice guideline update for the diagnosis, evaluation, prevention, and treatment of chronic kidney disease–mineral and bone disorder (CKD-MBD). Kidney Int Suppl. 2017;7:1–59. 10.1016/j.kisu.2017.04.001 PMC6340919 30675420

[9] Zhang H, Li G, Yu X, Yang J, Jiang A, Cheng H, et al. Progression of vascular calcification and clinical outcomes in patients receiving maintenance Dialysis. JAMA Netw Open. 2023;6:e2310909. 37126347 10.1001/jamanetworkopen.2023.10909 PMC10152309

[10] Kauppila LI, Polak JF, Cupples LA, Hannan MT, Kiel DP, Wilson PWF. New indices to classify location, severity and progression of calcific lesions in the abdominal aorta: a 25-year follow-up study. Atherosclerosis. 1997;132:245–50. 9242971 10.1016/s0021-9150(97)00106-8

[11] Cai Z, Liu Z, Zhang Y, Ma H, Li R, Guo S, et al. Associations between life’s essential 8 and abdominal aortic calcification among Middle-Aged and elderly populations. JAHA. 2023;12:e031146. 38063150 10.1161/JAHA.123.031146 PMC10863763

[12] Lewis JR, Schousboe JT, Lim WH, Wong G, Zhu K, Lim EM, et al. Abdominal aortic calcification identified on lateral spine images from bone densitometers are a marker of generalized atherosclerosis in elderly women. ATVB. 2016;36:166–73. 10.1161/ATVBAHA.115.306383 PMC4702255 26603153

[13] Lewis JR, Schousboe JT, Lim WH, Wong G, Wilson KE, Zhu K, et al. Long-Term atherosclerotic vascular disease risk and prognosis in elderly women with abdominal aortic calcification on lateral spine images captured during bone density testing: A prospective study. J Bone Miner Res. 2018;33:1001–10. 29443425 10.1002/jbmr.3405 PMC6415911

[14] Sethi A, Taylor DL, Ruby JG, Venkataraman J, Sorokin E, Cule M, et al. Calcification of the abdominal aorta is an under-appreciated cardiovascular disease risk factor in the general population. Front Cardiovasc Med. 2022;9:1003246. 36277789 10.3389/fcvm.2022.1003246 PMC9582957

[15] Qu H, Zhang S, Li X, Miao Y, Han Y, Ju R, et al. A deep learning model based on self-supervised learning for identifying subtypes of proliferative hepatocellular carcinoma from dynamic contrast-enhanced MRI. Insights Imaging. 2025;16:89. 40244356 10.1186/s13244-025-01968-w PMC12006648

[16] Akinci D’Antonoli T, Berger LK, Indrakanti AK, Vishwanathan N, Weiss J, Jung M, et al. TotalSegmentator MRI: robust Sequence-independent segmentation of multiple anatomic structures in MRI. Radiology. 2025;314:e241613. 39964271 10.1148/radiol.241613

[17] Dockès J, Varoquaux G, Poline J-B. Preventing dataset shift from breaking machine-learning biomarkers. GigaScience. 2021;10:giab055. 34585237 10.1093/gigascience/giab055 PMC8478611

[18] Wang K, Wang X, Xi Z, Li J, Zhang X, Wang R. Automatic segmentation and quantification of abdominal aortic calcification in lateral lumbar radiographs based on Deep-Learning-Based algorithms. Bioengineering. 2023;10:1164. 37892894 10.3390/bioengineering10101164 PMC10604574

[19] Reid S, Schousboe JT, Kimelman D, Monchka BA, Jafari Jozani M, Leslie WD. Machine learning for automated abdominal aortic calcification scoring of DXA vertebral fracture assessment images: A pilot study. Bone. 2021;148:115943. 33836309 10.1016/j.bone.2021.115943

[20] Verbeke F, Van Biesen W, Honkanen E, Wikström B, Jensen PB, Krzesinski J-M, et al. Prognostic value of aortic stiffness and calcification for cardiovascular events and mortality in Dialysis patients: outcome of the calcification outcome in renal disease (CORD) study. Clin J Am Soc Nephrol. 2011;6:153–9. 20829424 10.2215/CJN.05120610 PMC3022237

[21] Yi J, Wu P, Huang Q, Qu H, Metaxas DN. Vertebra-focused landmark detection for scoliosis assessment. In: 2020 IEEE 17th International Symposium on Biomedical Imaging (ISBI). Iowa City (IA): IEEE; 2020. pp. 736–40.

[22] He K, Zhang X, Ren S, Sun J. Deep residual learning for image recognition. In: 2016 IEEE Conference on Computer Vision and Pattern Recognition (CVPR). 2016. pp. 770–8.

[23] Graffy PM, Liu J, O’Connor S, Summers RM, Pickhardt PJ. Automated segmentation and quantification of aortic calcification at abdominal CT: application of a deep learning-based algorithm to a longitudinal screening cohort. Abdom Radiol. 2019;44:2921–8. 10.1007/s00261-019-02014-2 30976827

[24] Saleem A, Ilyas Z, Suter D, Hassan GM, Reid S, Schousboe JT, et al. SCOL: supervised contrastive ordinal loss for abdominal aortic calcification scoring on vertebral fracture assessment scans. 2023.

[25] Sharif N, Gilani SZ, Suter D, Reid S, Szulc P, Kimelman D, et al. Machine learning for abdominal aortic calcification assessment from bone density machine-derived lateral spine images. eBioMedicine. 2023;94:104676. 37442671 10.1016/j.ebiom.2023.104676 PMC10435763

[26] Dalla Via J, Gebre AK, Smith C, Gilani Z, Suter D, Sharif N, et al. Machine-Learning assessed abdominal aortic calcification is associated with Long-Term fall and fracture risk in Community-Dwelling older Australian women. J Bone Miner Res. 2023;38:1867–76. 37823606 10.1002/jbmr.4921 PMC10842308

[27] Ilyas Z, Saleem A, Suter D, Schousboe JT, Leslie WD, Lewis JR, et al. A hybrid CNN-Transformer feature pyramid network for granular abdominal aortic calcification detection from DXA images. In: Linguraru MG, Dou Q, Feragen A, Giannarou S, Glocker B, Lekadir K, et al. editors. Medical image computing and computer assisted Intervention – MICCAI 2024. Cham: Springer Nature Switzerland; 2024. pp. 14–25.

[28] Gilani SZ, Sharif N, Suter D, Schousboe JT, Reid S, Leslie WD, et al. Show, attend and detect: towards Fine-Grained assessment of abdominal aortic calcification on vertebral fracture assessment scans. In: Wang L, Dou Q, Fletcher PT, Speidel S, Li S, editors. Medical image computing and computer assisted Intervention – MICCAI 2022. Cham: Springer Nature Switzerland; 2022. pp. 439–50.

