# Supplementary material for: Automated abdominal aortic calcification scoring via deep learning: a multi-center validation of LVLCRNet
Source: BMC Med Imaging. 2025 Dec 1;26:4. doi: 10.1186/s12880-025-02072-7 (PMC12777190; doi:10.1186/s12880-025-02072-7)
Supplement: Supplementary file 1 — Supplementary Material 1 [file 12880_2025_2072_MOESM1_ESM.docx]

| Characteristic | Center A | Center B | Center C | Center D |
| --- | --- | --- | --- | --- |
| Lumbar lateral^a^ | 1108 (41.65%) | 556 (20.90%) | - | - |
| Vendor | Philips | Canon Inc. | - | - |
| Pixel spacing, mm | 0.14×0.14 | 0.125×0.125 | - | - |
| FOV, cm^2^ | 24.7×41.4 (20.7×31.0-40.5×42.8) | 20.3×41.5 (18.6×28.5-42.6×42.6) | - | - |
| Tube current^b^, mAs | 40 (25-65) | 64 (40-100) | - | - |
| Tube voltage^b^, KV | 77 (77-85) | 95 (85-105) | - | - |
| Abdominal lateral^a^ | 287 (10.79%) | 161 (6.05%) | 157 (5.90%) | 391 (14.70%) |
| Vendor | Philips | Canon Inc. | Philips | United film |
| Pixel spacing, mm | 0.14×0.14 | 0.125×0.125 | 0.147×0.147 | 0.131×0.131 |
| FOV, cm^2^ | 40.4×41.6 (31.6×36.7-43.3×43.3) | 36.9×41.5 (32.3×41.5-42.6×42.6) | 38.8×42.3 (30.1×35.9-42.3×43.2) | 37.9×43.2 (32.9×40.7-43.2×43.2) |
| Tube current^b^, mAs | 20 (11-20) | 64 (40-64) | 20 (20-70) | 16.5 (8-92) |
| Tube voltage^b^, KV | 77 (77-85) | 85 (85-91) | 77 (77-125) | 90 (77-97) |

**Table 1S** Summary of Imaging Acquisition Protocols and Equipment by Dataset.

aNumber of patients (%), bMedian (range)

FOV = field of view

**Table 2S** Differences in AACS Between Model Predictions and Manual Measurements.

|  | Test Sets | HRNet | LVLNet | LVLCRNet |
| --- | --- | --- | --- | --- |
| Mean | Internal Test Set | 0.62 | -0.13 | 0.13 |
|  | External Test Set 1 | 1.02 | 0.22 | 0.18 |
|  | External Test Set 2 | 0.32 | -0.29 | -0.25 |
| Median | Internal Test Set | 0.58 | 0.01 | 0.15 |
|  | External Test Set 1 | 1.20 | 0.32 | 0.19 |
|  | External Test Set 2 | 0.56 | -0.18 | -0.10 |

Note: differences = AACS_model_- AACS_GT_.

Table 3S. Results of the TOST for AACS between the ground truth and the three models.

| Parameters | Internal Test (n = 211) | | | External Test 1 (n = 157) | | | External Test 2 (n = 391) | | | |
| --- | --- | --- | --- | --- | --- | --- | --- | --- | --- | --- |
|  | HRNet | LVLNet | LVLCRNet | HRNet | LVLNet | LVLCRNet | HRNet | LVLNet | LVLCRNet |  |
| Difference (Mean ± SD) | 0.61 ± 2.66 | -0.13 ± 2.14 | 0.13 ± 2.01 | 1.02 ± 2.45 | 0.22 ± 2.09 | 0.18 ± 2.03 | 0.32 ± 3.11 | -0.29 ± 2.49 | -0.25 ± 2.39 |  |
| Difference (95%CI) | [0.25, 0.98] | [-0.42, 0.16] | [-0.14, 0.40] | [0.63, 1.40] | [-0.11, 0.55] | [-0.14, 0.50] | [0.01, 0.63] | [-0.54, -0.05] | [-0.49, -0.01] |  |
| +1 (+Δ) | t = -2.11,  *p* = 0.018 | t = -7.68,  *p* <0.001 | t = -6.28,  *p* <0.001 | t = 0.09,  *p* = 0.54 | t = -4.69,  *p* <0.001 | t = -5.07,  *p* <0.001 | t = -4.34,  *p* <0.001 | t = -10.29,  *p* <0.001 | t = -10.34，  *p* <0.001 |  |
| -1 (-Δ) | t = 8.81,  *p* <0.001 | t = 5.91,  *p* <0.001 | t = 8.14,  *p* <0.001 | t = 10.33  *p* <0.001 | t = 7.31  *p* <0.001 | t = 7.29  *p* <0.001 | t = 8.38  *p* <0.001 | t = 5.62  *p* <0.001 | t = 6.17  *p* <0.001 |  |
| Final *p* value | *p* = 0.018 | *p* <0.001 | *p* <0.001 | *p* = 0.54 | *p* <0.001 | *p* <0.001 | *p* <0.001 | *p* <0.001 | *p* <0.001 |  |
| Equivalence | Yes | Yes | Yes | No | Yes | Yes | Yes | Yes | Yes |  |
| +0.5 (+Δ) | t = 0.62,  *p* = 0.73 | t = -4.29,  *p* <0.001 | t = -2.67,  *p* = 0.004 | t = 2.65,  *p* = 1.00 | t = -1.69,  *p* = 0.05 | t = -1.98,  *p* = 0.02 | t = -1.16,  *p* = 0.12 | t = -6.31,  *p* <0.001 | t = -6.21,  *p* <0.001 |  |
| -0.5 (-Δ) | t = 6.08,  *p* <0.001 | t = 2.51  *p* = 0.006 | t = 4.54  *p* <0.001 | t = 7.77  *p* <0.001 | t = 4.31  *p* <0.001 | t = 4.20  *p* <0.001 | t = 5.20  *p* <0.001 | t = 1.64  *p* = 0.05 | t = 2.04  *p* = 0.02 |  |
| Final *p* value | *p* = 0.73 | *p* = 0.006 | *p* = 0.004 | *p* = 1.00 | *p* = 0.05 | *p* = 0.02 | *p* = 0.12 | *p* = 0.05 | *p* = 0.02 |  |
| Equivalence | No | Yes | Yes | No | No | Yes | No | No | Yes |  |

Difference =AACS_model_- AACS_GT_

TOST = Two-One-Sided Tests, SD = standard deviation, CI = confidence interval, AACS = abdominal aortic calcification score, GT = ground truth.


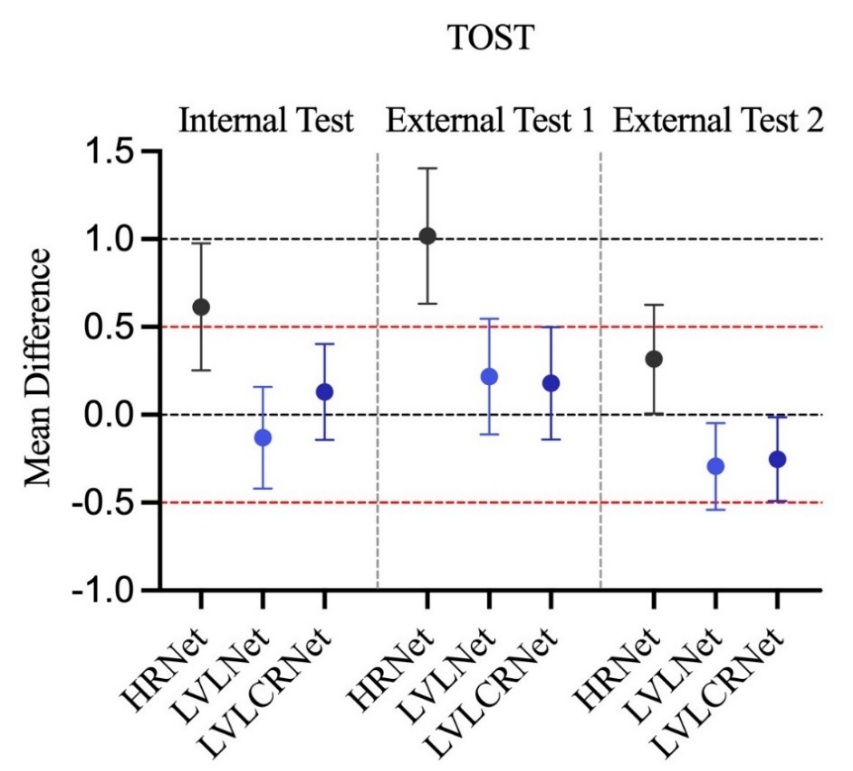


Fig. 1S. Results of the TOST for AACS between the ground truth and the three models.
